# Supplementary material for: Hybrid Models and Biological Model Reduction with PyDSTool
Source: PLoS Comput Biol. 2012 Aug 9;8(8):e1002628. doi: 10.1371/journal.pcbi.1002628 (PMC3415397; doi:10.1371/journal.pcbi.1002628)
Supplement: Text S4 — Complete source code for the PyDSTool package (version 0.88.120504). Includes API documentation and help files linking to web pages. This file is identical to the current public release on Sourceforge.net. (ZIP) [file pcbi.1002628.s004.zip › PyDSTool/html/toc-PyDSTool.PyCont.ContClass'-module.html]

xml version="1.0" encoding="ascii"?


ContClass'


# Module ContClass'

---

## Classes

ContClass  

## Functions

beta

binomial

bytes

chisquare

distutil\_destination

exponential

f

gamma

geometric

get\_state

gumbel

hypergeometric

laplace

logistic

lognormal

logseries

multinomial

multivariate\_normal

negative\_binomial

noncentral\_chisquare

noncentral\_f

normal

pareto

permutation

poisson

power

rand

randint

randn

random

random\_integers

random\_sample

ranf

rayleigh

sample

seed

set\_state

shuffle

standard\_cauchy

standard\_exponential

standard\_gamma

standard\_normal

standard\_t

triangular

uniform

vonmises

wald

weibull

zipf

## Variables

ALLOW\_THREADS

BUFSIZE

CLIP

DAILY

ERR\_CALL

ERR\_DEFAULT

ERR\_DEFAULT2

ERR\_IGNORE

ERR\_LOG

ERR\_PRINT

ERR\_RAISE

ERR\_WARN

FLOATING\_POINT\_SUPPORT

FPE\_DIVIDEBYZERO

FPE\_INVALID

FPE\_OVERFLOW

FPE\_UNDERFLOW

FR

False\_

HOURLY

Inf

Infinity

MAXDIMS

MINUTELY

MO

MONTHLY

NAN

NINF

NZERO

NaN

PINF

PZERO

RAISE

SA

SECONDLY

SHIFT\_DIVIDEBYZERO

SHIFT\_INVALID

SHIFT\_OVERFLOW

SHIFT\_UNDERFLOW

SU

ScalarType

TH

TU

True\_

UFUNC\_BUFSIZE\_DEFAULT

UFUNC\_PYVALS\_NAME

WE

WEEKLY

WRAP

YEARLY

\_classes

\_constants

\_pydstool\_path

absolute

add

arccos

arccosh

arcsin

arcsinh

arctan

arctan2

arctanh

auto\_list  

bitwise\_and

bitwise\_not

bitwise\_or

bitwise\_xor

c\_

cast

ceil

colorbar\_doc

conj

conjugate

copysign

cos

cosh

curve\_args\_list  
 curve\_list  

deg2rad

degrees

divide

e

equal

exp

exp2

expm1

fabs

floor

floor\_divide

fmax

fmin

fmod

frexp

greater

greater\_equal

hypot

index\_exp

inf

infty

invert

isfinite

isinf

isnan

ldexp

left\_shift

less

less\_equal

little\_endian

log

log10

log1p

logaddexp

logaddexp2

logical\_and

logical\_not

logical\_or

logical\_xor

maximum

mgrid

minimum

mod

modf

multiply

nan

nbytes

negative

newaxis

nextafter

not\_equal

ogrid

ones\_like

pi

r\_

rad2deg

radians

rcParams

rcParamsDefault

reciprocal

remainder

rerr

right\_shift

rint

rout

s\_

sctypeDict

sctypeNA

sctypes

sign

signbit

sin

sinh

spacing

sqrt

square

subtract

tan

tanh

true\_divide

trunc

typeDict

typeNA

typecodes

---

[hide private]
